# Vulvovaginal Candidiasis Among Patients Attending the Gynecology Outpatient Department (GOPD) and STD Clinic With Special Reference to Their Antifungal Susceptibility and the Genetic Diversity of Non-albicans Candida in a Tertiary Care Hospital

**DOI:** 10.7759/cureus.105579

**Published:** 2026-03-21

**Authors:** Suranjana C Hazarika, Lakshyasri Baishya, Ridip Dutta, Kishore Sarma, Monjuri Chakravarty, Himanto N Hazarika

**Affiliations:** 1 Department of Microbiology, Gauhati Medical College and Hospital, Guwahati, IND; 2 Department of Computational Biology and Biotechnology, Mahapurusha Srimanta Sankaradeva Viswavidyalaya, Guwahati, IND; 3 Department of Ophthalmology, Nalbari Medical College and Hospital, Nalbari, IND

**Keywords:** antifungal susceptibility testing, candida albicans, genetic diversity, non-albicans candida species, vulvovaginal candidiasis

## Abstract

Background

Vulvovaginal candidiasis (VVC) is a common opportunistic infection worldwide. This study, undertaken at a tertiary care center in North-East India, aimed to assess VVC among clinically suspected cases. The study also determined the epidemiological distribution of the associated *Candida* species (spp.), along with an evaluation of their antifungal susceptibility patterns.

Methods

Specimens from 300 clinically suspected cases of VVC were subjected to phenotypic and molecular identification methods, along with antifungal susceptibility testing by disk diffusion test and minimum inhibitory concentration (MIC) determination.

Results

Ninety-six cases (32%) showed the growth of *Candida* species. The mean age of culture-positive cases was 29.6 years. VVC was found to be more common among married women (85.42%) than in unmarried women (13.54%). *Candida albicans *(*C. albicans*)* *was the predominant pathogen, accounting for 75% (n = 72) of the total isolates, and the rest (25%, n = 24) were non-albicans* Candida *(NAC)* *species, dominated by *C. tropicalis* (15.63%) and followed by* C*. *glabrata *(4.17%), *Pichia kudriavzevii *(*P. kudriavzevii*)* *(3.13%), and *C. parapsilosis* (2.08%). A high degree of drug resistance was seen to most of the azoles, especially fluconazole. Voriconazole displayed a lower degree of resistance, besides nystatin and amphotericin B, and can thus be used with confidence for the treatment of VVC in this region.

Conclusion

Though *Candida albicans *is the predominant species, non-albicans *Candida*,* *such as *Candida tropicalis *and *Candida glabrata*,* *are being increasingly reported in VVC, showing high resistance to azole antifungals. The prompt identification of Candida isolates and the determination of antifungal sensitivity patterns will help to improve treatment outcomes for VVC.

## Introduction

In recent years, fungi have emerged as significant causes of morbidity and mortality, particularly among immunocompromised and critically ill patients. A variety of *Candida* species (spp.) are responsible for the most common opportunistic fungal infections [1]. The alarming rise in multidrug-resistant bacterial infections has led to the excessive use of broad-spectrum antibiotics, which disrupt normal microbial flora and promote the overgrowth of *Candida* species, thereby increasing their potential to cause disease [2].

Vulvovaginal candidiasis (VVC) is a common fungal infection of the female genital tract caused by the overgrowth of *Candida* species, most frequently *Candida albicans *(*C. albicans*). It is better described as a sexually associated condition rather than a classical sexually transmitted disease and may be caused by different *Candida* spp. [3]. VVC is an inflammatory condition of the vulva and vagina and is considered the second most common cause of vaginal infection after bacterial vaginosis [4-6]. Clinical manifestations are often nonspecific and typically include pruritus, vaginal discharge, tenderness, irritation, and burning sensations, which may result in dyspareunia and dysuria [7-9].

The global significance of VVC stems from its high prevalence, association with sexually transmitted infections, potential to cause ascending genital tract infections, and both direct and indirect economic burden [10,11]. Although VVC is not directly life-threatening, it can lead to significant physical discomfort, psychological distress, recurrent infections, and substantial economic burden. Despite advances in antifungal therapy, VVC remains a global health concern, and its pathogenic mechanisms are not yet fully understood [12].

*Candida albicans* accounts for approximately 85% of VVC cases in India, while the remaining 15% are attributed to non-albicans *Candida* (NAC) species [13]. The infection process begins with the adhesion of *Candida* to vaginal epithelial cells, followed by the proliferation and induction of symptomatic inflammation. In addition to *C. albicans*, other species such as *Candida glabrata*, *Candida tropicalis*, *Candida parapsilosis*, and *Pichia kudriavzevii* (*Candida krusei*) have been implicated in VVC [14,15]. VVC is categorized as uncomplicated or complicated. Uncomplicated VVC involves fewer than four episodes per year, presents with mild to moderate symptoms, and is usually caused by *C. albicans* in otherwise healthy women. Complicated VVC includes infections caused by non-albicans species, recurrent episodes, or infections occurring in women with underlying risk factors [16,17]. Several predisposing factors contribute to the development of VVC, including sociodemographic variables, pregnancy, uncontrolled diabetes, the prolonged use of broad-spectrum antibiotics, oral contraceptive use, sexual activity, poor personal hygiene, hormone replacement therapy, immune deficiencies, and genetic susceptibility [7,17,18].

Accurate species identification is essential because virulence, epidemiology, and susceptibility to antifungal agents vary among different *Candida* species. Recent studies have shown that antifungal resistance can emerge through intrinsic or acquired mechanisms, with long-term antifungal therapy being a major contributor to acquired resistance [18,19]. Therefore, understanding the antifungal susceptibility profiles of *Candida* species is crucial for guiding appropriate treatment decisions in VVC.

Despite the clinical significance of VVC, there is limited epidemiological data regarding the distribution of *Candida* species isolated from VVC cases in the north-east region of India. Additionally, emerging evidence suggests geographic heterogeneity in the distribution and resistance patterns of *Candida* species. A clearer understanding of regional species prevalence, antifungal susceptibility profiles, and genetic diversity is therefore necessary.

In the present study, our primary objectives were to determine the occurrence of different *Candida* spp. among clinically suspected cases of VVC and evaluate their antifungal susceptibility profiles. We also examined the genetic diversity of non-albicans *Candida* species isolated in our study.

## Materials and methods

Study design

This prospective cross-sectional study was carried out over a period of two years, from March 2023 to March 2025, at Gauhati Medical College and Hospital, a tertiary care hospital located in Guwahati, Assam, which is a state in the north-east region of India.

Patient selection

Three hundred clinically suspected cases of vulvovaginal candidiasis belonging to the reproductive age group, but nonpregnant, were taken up for the study. The cases were recruited from the STD clinic and the gynecology OPD of Gauhati Medical College and Hospital. Clinically suspected cases presenting with symptoms of vulvovaginitis, such as vulvar burning, pruritus vulvae, dyspareunia, vaginal soreness and irritation, pain or discomfort during urination, and abnormal vaginal discharge, were selected for further examination. The patients who gave a history of taking antifungals in the last three months were excluded from the study. All asymptomatic patients, those not belonging to the reproductive age group, and those not giving consent were excluded from the study.

Sample size calculation

Sample size was calculated based on the number of cases of vulvovaginitis that were reported from the STD clinic and the gynecology OPD in the previous three years.

Ethical considerations

Patients were briefed on the protocol, and after voluntarily agreeing to participate in the study, they signed an informed consent form. The study was initiated after receiving ethical clearance from the Institutional Ethical Committee of Gauhati Medical College and Hospital under the registration number MC/190/2007/Pt-II/March-2003/5, dated 10/04/2023.

Sample size calculation

A retrospective review of hospital records over the past three years was conducted to estimate the prevalence of *Candida* in VVC for sample size calculation, and the prevalence was found to be 26.5%.

The sample size was calculated using the single population proportion formula N = Z^2^p(1 - p) / d^2^. Assuming the estimated prevalence (p) = 26.5% (0.265), the complement of prevalence (q) = 1 - 0.265 = 0.735, 95% confidence level (Z) = 1.96, and the margin of error (d) = 5% (0.05), the sample size is therefore 299.24 participants, which was rounded off to 300 participants.

Sample collection

Two swab specimens of pooled vaginal discharge from the posterior fornix were collected with sterile cotton swabs, or scrapings were taken from the vagina and edges of erythematous lesions of the vulva with a cotton wool swab soaked in saline [20].

Laboratory procedures

Preliminary microscopic examination was done by the preparation of a potassium hydroxide (KOH) wet mount. Specimens were examined with 10% freshly prepared KOH solution under a 40× objective to identify the presence of round or oval budding yeast cells, along with the presence of mycelia or pseudohyphae.

Culture

*Candida* culture was done in Sabouraud dextrose agar (SDA) with gentamicin and incubated at 37°C for 48 hours. The growth of colonies appearing pasty, opaque, slightly domed or flat, smooth, and pale-colored (white, off-white, or beige) with a sweet smell reminiscent of ripe apples was suspected to be colonies of *Candida* [21]. Suspected colonies of *Candida* isolates were further tested by Gram stain, germ tube test, etc.

The phenotypic identification of *Candida* species was done by the germ tube test, the study of chlamydospore and blastospore formation on Corn Meal Tween Agar culture according to Giri and Kindo [21]. Germ tube-negative *Candida* isolates were classified on the basis of the sugar assimilation test and colony color on Hichrom Candida agar. HiCandida Identification Kit (HiMedia Laboratories Pvt. Ltd., Mumbai, India) supplemented the identification of isolates.

Antifungal susceptibility testing

In vitro antifungal susceptibility testing of *Candida* isolates was performed using the disk diffusion method with antifungal disks (HiMedia Laboratories Pvt. Ltd., Mumbai, India), as per instructions provided by the manufacturer and also as per laboratory guidelines. As per the “Clinical and Laboratory Standards Institute (CLSI)” (M44-A) guidelines, the inoculum was prepared by selecting five particular colonies from a 24-hour-old culture of *Candida* species. Colonies were then suspended in 5 mL of sterile 0.9% normal saline [22]. The suspension was then vortexed to get uniform turbidity and adjusted visually to 0.5 McFarland standards. After 15 minutes, the suspension was inoculated onto “Mueller Hinton Glucose Agar (MHGA)” with 2% glucose and 0.5 µg/mL of methylene blue. Antifungal disks used include fluconazole (10 µg), clotrimazole (10 µg), nystatin (100 U), voriconazole (1 µg), miconazole (30 µg), and amphotericin B (20 µg) (HiMedia Laboratories Pvt. Ltd., Mumbai, India). Antifungal disks were placed evenly, keeping a distance of 24 mm from center to center. The plates were then placed in an incubator at 37°C for 24-48 hours. *Candida albicans* ATCC 90028, *Candida tropicalis* ATCC 750, *Candida krusei *ATCC 6258, and *Candida parapsilosis* ATCC 22019 were used as controls [23-25].

The minimum inhibitory concentration (MIC) was determined using the bioMérieux E-test method as per the manufacturer’s instructions. The antifungals tested for MIC include fluconazole (range: 0.016-256 µg/mL), voriconazole (range: 0.002-32 µg/mL), and amphotericin B (0.002-32 µg/mL). The MIC of each drug was determined after incubation for 48 hours at 35℃ in moist conditions. The results of the antifungal susceptibility test were interpreted as sensitive (S), intermediate sensitive (I), or susceptible dose dependent (SDD) and resistant (R) based on zone diameter in mm. Interpretative criteria for azoles are as recommended by the Clinical and Laboratory Standards Institute (CLSI) [22].

Molecular analyses

DNA Extraction

Genomic DNA from the cultured fungal specimen was extracted using the Wizard® Genomic DNA Purification Kit (Promega Corporation, Madison, WI) following the manufacturer’s instructions with slight modification. Fungal colonies were inoculated in a glass tube containing Yeast Peptone Dextrose (YPD) medium and incubated at 30°C overnight in a shaker incubator; 1 mL aliquot of 20 hour-incubated YPD broth was centrifuged, and the pellet was further incubated for one hour at 37°C with 600 µL mixture of 1 M sorbitol, 0.1 M EDTA, 0.1% (w/v) zymolyase-100T (G-bioTM), and 1% (v/v) 2-mercaptoethanol (G-bioTM) adjusted to a pH of 7.5. Cell lysis was carried out with Proteinase K (Promega Corporation, Madison, WI) and cell lysis buffer at 56°C. The subsequent steps followed the manufacturer’s protocol [26]. The purity and concentration of extracted DNA were measured using a NanoDrop spectrophotometer (Eppendorf®, Hamburg, Germany), and DNA was stored at -20°C for further downstream analysis.

Primer Designing

Universal primers targeting the fungal internal transcribed spacer (ITS) region of the ribosomal RNA gene were adopted from published research papers and validated using the NCBI primer Basic Local Alignment Search Tool (BLAST) tool (Bethesda, MD) [27]. Moreover, an in silico polymerase chain reaction (PCR) analysis was performed to assess GC content, melting temperature (Tm) value, hair loop formation, and the probability of primer dimer formation. Quality-validated primers (desalted oligos) were synthesized by IDT Corporation, Singapore.

PCR Amplification

The ITS1 and ITS4 regions were amplified with conventional PCR using GoTaq Mastermix (Promega® Corporation, Madison, WI) as per the manufacturer’s instructions.

The amplification of the ITS regions was performed using the following cycling conditions: initial denaturation at 95°C for five minutes, 30 cycles of denaturation at 95°C for one minute, primer annealing at 56°C for one min, and extension at 72°C for one minute, followed by a final extension step at 72°C for five minutes. Samples showing weak amplification were further amplified with the inner primer pair following the nested PCR protocol. The PCR products were analyzed on 1.2% agarose gel electrophoresis and visualized under UV.

PCR Product Purification

The PCR products were examined for integrity and specificity. Based on the requirement, the PCR amplicons were subjected to low-voltage agarose gel electrophoresis. Well-resolved bands were excised and purified using a gel purification kit (NucleoSpin®, Takara, Kyoto, Japan).

DNA sequencing

All PCR-positive samples were subjected to Sanger sequencing at Apical Scientific Sdn. Bhd., Selangor, Malaysia, using an Applied Biosystems ABI PRISM 3730xl Genetic Analyzer with the BigDye™ Terminator v3.1 Cycle Sequencing Kit (Thermo Fisher Scientific, Waltham, MA). Raw chromatograms (.ab1 files) were processed in BioEdit version 7.2.5, where low-quality regions were trimmed and ambiguous bases manually corrected [28]. Forward and reverse reads were assembled to generate consensus sequences. A total of 29 high-quality sequences were obtained and verified for species identification using NCBI BLAST before submission to the NCBI GenBank database. Subsequent phylogenetic analyses were performed using MEGA X [29].

A subset of isolates were selected for molecular sequencing due to cost constraints. All 24 of the non-albicans *Candida* isolates, as well as five of the *Candida albicans* isolates, were selected for sequencing. A simple random sampling technique was employed to select the five *Candida albicans* isolates from a total population of 72 using Microsoft Excel’s RAND() function (Microsoft Corp., Redmond, WA), ensuring that each unit had an equal probability of selection.

Phylogenetic tree construction

A total of 79 nucleotide sequences were included in the phylogenetic analysis, comprising 29 sequences generated in this study and 50 reference sequences representing major *Candida* species retrieved from public databases. Multiple sequence alignment was performed using the ClustalW algorithm implemented in MEGA X, followed by manual inspection and the trimming of ambiguous regions. Phylogenetic relationships were inferred using the maximum likelihood (ML) method with the Kimura 2-parameter substitution model, selected based on the Bayesian information criterion (BIC). Rate heterogeneity among sites was modeled using a discrete gamma distribution with five rate categories (+G) and a shape parameter of 0.8921. Branch support was assessed using 500 bootstrap replicates, and branch lengths were expressed as substitutions per site.

Tree visualization

The ML tree generated in MEGA X was exported in Newick format and visualized using the Interactive Tree of Life (iTOL). Study isolates were highlighted in red, while reference sequences were color-coded according to species complexes.

## Results

Of the 300 women examined for vulvovaginal candidiasis, 96 (32%) women showed growth of *Candida* species. All cases showed the growth of single isolates, and mixed infection of *Candida *species was not seen in any of the 96 cases of positive fungal culture. The mean age of the cases with a positive culture for *Candida* species was 29.6 years, ranging from 18 to 54 years. A total of 96 cases of vulvovaginal candidiasis were included in the chi-square (χ²) analysis after combining age groups with sparse counts. The distribution of cases differed significantly from a uniform distribution across age categories (χ² = 34.33; degrees of freedom {df} = 3; p < 0.001). The highest incidence was observed in women aged 26-35 years (40 cases), followed by 18-25 years (36 cases), while lower frequencies were seen in the 36-45-year (14 cases) and 46-55-year (six cases) age groups. These findings indicate that vulvovaginal candidiasis predominantly affects women of reproductive age (Table 1).

**Table 1:** Age distribution of the 96 cases of vulvovaginal candidiasis

Age Group (in Years)	Number of Cases of Vulvovaginal Candidiasis
<18	0
18-25	36
26-35	40
36-45	14
46-55	6
≥56	0

Among the 300 clinically suspected cases of vulvovaginal candidiasis (VVC), the most frequently reported symptom was vulvovaginal itching (62.5%), followed closely by vaginal discharge (61.4%) and soreness/irritation in the vaginal area (57.2%). Dyspareunia was reported by 48.9% of the participants, while dysuria was the least common symptom (30.2%). The high frequency of itching and discharge is consistent with the classic clinical presentation of VVC, reflecting inflammatory changes of the vulvovaginal epithelium and the presence of curd-like secretions typically associated with *Candida* infection.

It is important to clarify that these symptoms are not mutually exclusive; rather, they frequently overlap in the same patient. A single individual may present with itching, discharge, and soreness simultaneously, as these manifestations arise from the same underlying inflammatory process. Therefore, the cumulative percentages exceed 100%, indicating co-occurrence rather than independent symptom categories. Dyspareunia and dysuria may also coexist with primary symptoms due to mucosal inflammation and epithelial microabrasions (Table 2).

**Table 2:** Frequency of symptoms (out of a total of 96 cases)

Value	Frequency	Percent (%)
Vulvovaginal itching	60	62.5%
Vaginal discharge	59	61.4%
Soreness and irritation in the vaginal area	55	57.2%
Dyspareunia	47	48.9%
Dysuria	29	30.2%

The distribution of culture positivity according to marital status is shown in Table 3.

**Table 3:** Association of marital status with Candida culture positivity in VVC VVC: vulvovaginal candidiasis

Marital Status	Culture-Positive	Culture-Negative	Total
Married	82 (43.9%)	105 (56.1%)	187
Unmarried	13 (11.8%)	97 (88.2%)	110
Widow	1 (100%)	0 (0%)	1
Divorced	0 (0%)	2 (100%)	2
Total	96 (32%)	204 (68%)	300

Married women constituted 187 (62.3%) of the study population, of whom 82 (43.9%) were culture-positive. Among 110 (36.7%) unmarried women, 13 (11.8%) were culture-positive. One widow was included in the study and was culture-positive (100%), while none of the two divorced women were culture-positive.

A statistically significant association was observed between marital status and *Candida* culture positivity (χ² = 39.93; degrees of freedom {df} = 3; p < 0.001). Because some expected cell counts were less than five, Fisher’s exact test was applied, which also demonstrated a statistically significant association (p < 0.001).

When married women were compared to unmarried women, the odds of culture positivity were significantly higher among married women (odds ratio {OR} = 5.82; 95% confidence interval {CI}: 3.08-10.99; p < 0.001).

Identification of *Candida* species

A total of 96 *Candida* isolates were obtained from patients with vulvovaginal candidiasis. The majority of isolates belonged to *Candida albicans* (72/96, 75%), while non-albicans *Candida* species accounted for 24 (25%) isolates. Among the non-albicans *Candida* species, the most frequently isolated organism was *Candida tropicalis* (15/96, 15.6%), followed by *Candida glabrata* (4/96, 4.2%), *Candida parapsilosis* (2/96, 2.1%), and *Pichia kudriavzevii *(*P. kudriavzevii*) (previously *Candida krusei*) (3/96, 3.1%). Statistical analysis using the chi-square test demonstrated a significant predominance of *Candida albicans* compared to non-albicans *Candida* species among the isolates (χ² = 24.0; df = 1; p < 0.001) (Table 4).

**Table 4:** Occurrence and frequency of isolates

Isolate (n = 96)	Frequency	Percentage (%)
Candida albicans	72	75%
Candida tropicalis	15	15.63%
Candida glabrata	4	4.17%
Pichia kudriavzevii	3	3.13%
Candida parapsilosis	2	2.08%

**Figure 1:**
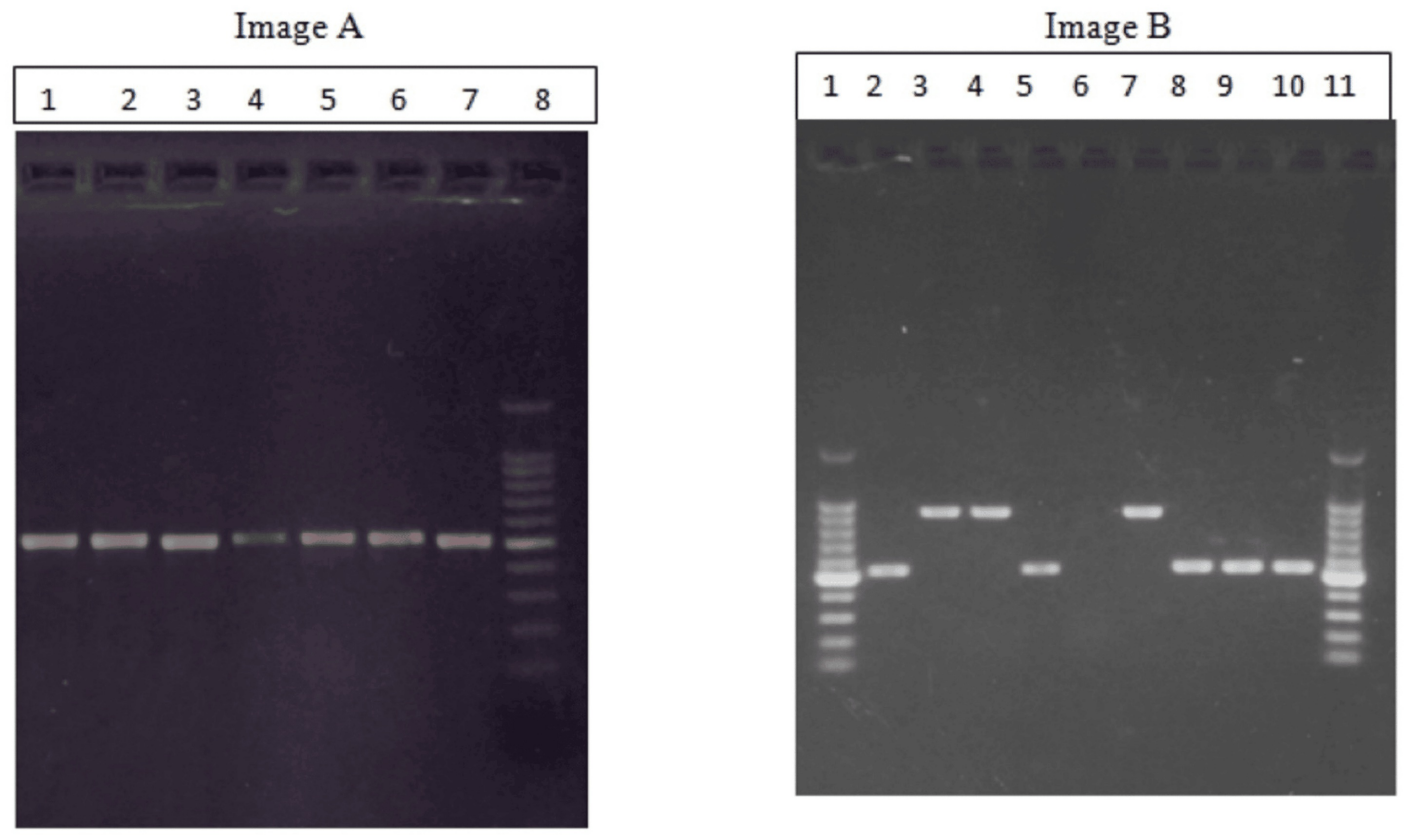
Gel images of ITS gene region-targeted PCR products on 1.2% agarose under UV (A) Well 1-7, positive bands for the ITS region in position 530 bp; well 8, DNA ladder. (B) Well 1 and 11, DNA ladder; well 2, 5, 8, 9, and 10, positive for ITS with size ~580 bp; and well 3, 4, and 7, positive for ITS with size ~910 bp PCR, polymerase chain reaction; bp, base pairs; ITS, internal transcribed spacer

Molecular characterization was done by PCR for the amplification of the ITS1 and ITS4 regions of the *Candida* isolates (Figure 1).

DNA sequencing was performed on PCR products to characterize the isolates at the molecular level and assess their genetic diversity. All 24 non-albicans *Candida* isolates and five *Candida albicans* isolates were subjected to sequencing. The maximum likelihood (ML) phylogenetic tree revealed clearly separated clades corresponding to major *Candida* and related yeast species. Bootstrap values at deeper nodes were high, providing statistical support for the inferred evolutionary relationships. The clustering pattern observed in the tree was consistent with established species-level taxonomy.

**Figure 2:**
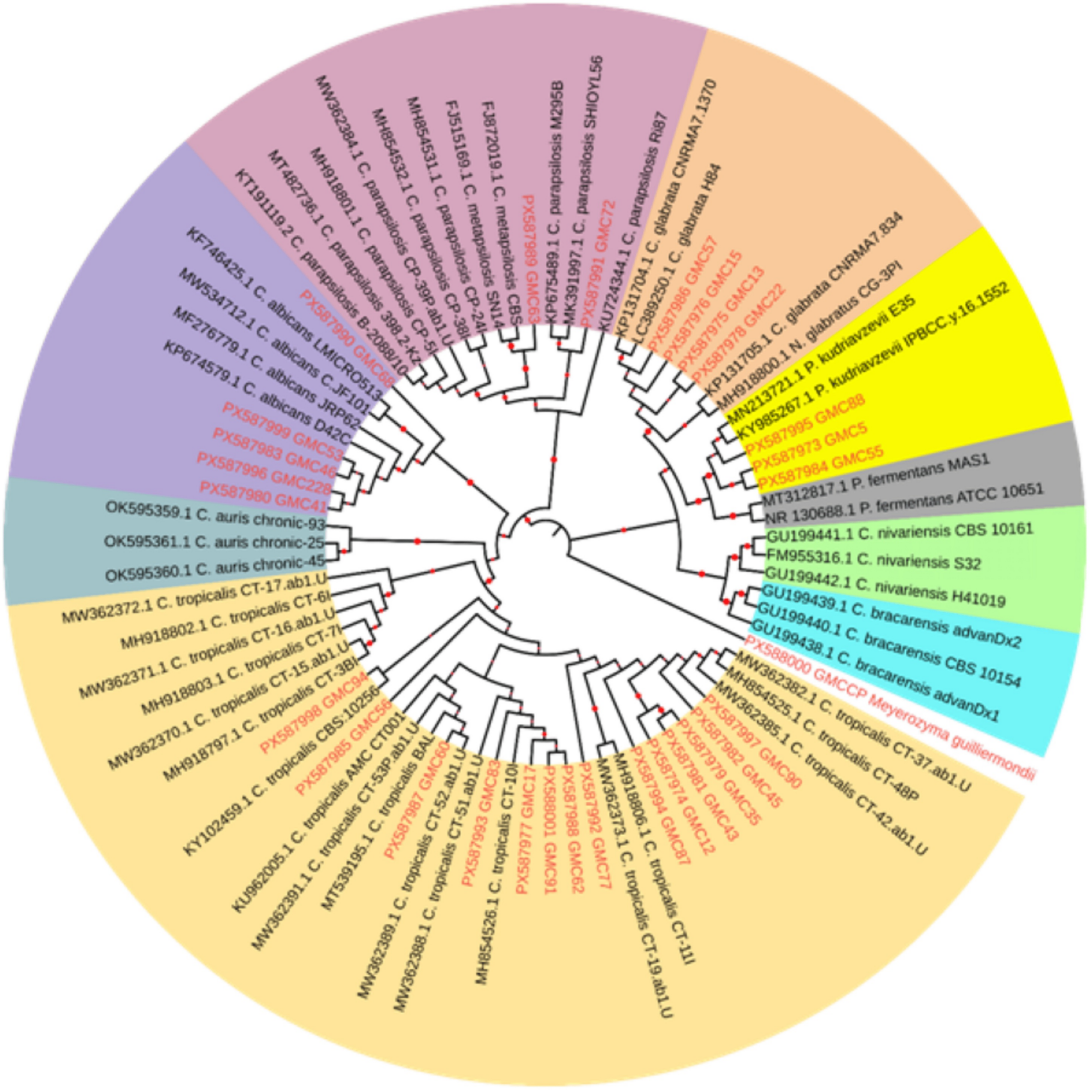
The tree illustrates the evolutionary relationships among 29 isolates obtained in the present study (highlighted in red) and 50 reference sequences representing major Candida species complexes Evolutionary history was inferred using the maximum likelihood method with the K2P model, incorporating a discrete gamma distribution (+G, shape parameter = 0.8921) to account for rate variation among sites. The phylogenetic tree was generated in MEGA X and visualized using the Interactive Tree of Life (iTOL) platform for enhanced annotation and color-coded clade representation K2P: Kimura 2-parameter

In total, 29 newly generated isolates (highlighted in red) were distributed across several species-level clades (Figure 2). A majority of the isolates were clustered within the *C. tropicalis* lineage, forming several closely related subgroups. Five isolates were grouped with the *C. albicans* clade. Four isolates were clustered with *C. glabrata*, while three isolates were grouped within the *Pichia kudriavzevii* (formerly *Candida krusei*) clade, each clustering with corresponding reference sequences. Two isolates were placed within the *C. parapsilosis* complex.

Most isolates exhibited relatively short terminal branches, whereas a few isolates within the *C. tropicalis* and *C. glabrata* clusters showed comparatively longer branches in the phylogenetic tree.

The susceptibility testing of *Candida* isolates revealed variable responses to antifungal agents (Table 5). Among *Candida albicans* (n = 72), sensitivity to fluconazole was observed in 52.78% of the isolates, with 8.33% showing susceptible dose dependent (SDD) response and 44.44% demonstrating resistance. The higher susceptibility of *C. albicans* was noted for clotrimazole (76.39%), nystatin (86.11%), voriconazole (88.89%), and miconazole (80.56%). Among *Candida tropicalis* (n = 15), sensitivity to fluconazole was 53.33%, while 20% were SDD, and 33.33% were resistant. The susceptibility of *C. tropicalis* to clotrimazole, nystatin, voriconazole, and miconazole was 60%, 66.67%, 80%, and 60%, respectively. *Candida glabrata* (n = 4) showed high resistance to fluconazole (75%), with variable susceptibility to clotrimazole (25%), nystatin (50%), and voriconazole (50%). *Pichia kudriavzevii* (n = 3) demonstrated no sensitivity to fluconazole, with 66.67% resistance, while better susceptibility was observed with nystatin (66.67%). Both isolates of *Candida parapsilosis* (n = 2) were sensitive to nystatin and voriconazole, whereas clotrimazole showed 50% resistance. Overall, higher susceptibility was observed for nystatin and voriconazole, while comparatively higher resistance was noted with fluconazole, particularly among non-albicans *Candida *species. All isolates of *Candida albicans* (n = 72), *Candida tropicalis* (n = 15), *Candida glabrata* (n = 4), *Pichia kudriavzevii *(n = 3), and *Candida parapsilosis *(n = 2) were sensitive to amphotericin B.

**Table 5:** Antifungal susceptibility patterns of isolates (n = 96) S, sensitive; I, intermediate or susceptible dose dependent (SDD); R, resistant

Antifungal Agent	*Candida albicans* (n = 72)			*Candida tropicalis* (n = 15)			*Candida glabrata* (n = 4)			*Pichia kudriavzevii* (n = 3)			*Candida parapsilosis* (n = 2)		
	S	I	R	S	I	R	S	I	R	S	I	R	S	I	R
Fluconazole	38	6	32	8	3	5	1	0	3	0	1	2	1	0	1
Clotrimazole	55	5	12	9	2	4	1	1	2	0	2	1	1	0	1
Nystatin	62	0	10	10	1	4	2	1	1	2	0	1	2	0	0
Voriconazole	64	2	6	12	0	3	2	1	1	2	0	1	2	0	0
Miconazole	58	2	12	9	1	5	1	0	3	0	2	1	1	0	1
Amphotericin B	72	0	0	15	0	0	4	0	0	3	0	0	2	0	0

The minimum inhibitory concentrations (MICs) of fluconazole, voriconazole, and amphotericin B for the isolates of the different species of *Candida* are given in Table 6. For quality control, the reference strain was tested simultaneously with the *Candida* isolates, and the quality control ranges of the reference strain *Candida albicans* ATCC 90028 are shown in Table 7.

**Table 6:** MICs (µg/mL) of antifungal agents for 96 isolates by E-test after 24 hours *The range of MIC values observed among the isolates indicates concentrations of the drugs that inhibited the most susceptible isolate and the least susceptible (more resistant) isolate among the different species of Candida isolated in the study MICs: minimum inhibitory concentrations

*Candida* Species	Antifungal Agent	MICs (µg/mL) by E-test*
*C. albicans* (n = 72)	Fluconazole	0.64-16
	Voriconazole	0.024-1.08
	Amphotericin B	0.15-0.75
*C. tropicalis* (n = 15)	Fluconazole	0.12-9
	Voriconazole	0.006-1.44
	Amphotericin B	0.125-0.5
*C. glabrata* (n = 4)	Fluconazole	0.52-32
	Voriconazole	0.84-2
	Amphotericin B	0.25-0.5
*P. kudriavzevii* (n = 3)	Fluconazole	0.36-12
	Voriconazole	0.75-1.8
	Amphotericin B	0.35-0.7
*C. parapsilosis* (n = 2)	Fluconazole	1.2-12
	Voriconazole	0.08-0.5
	Amphotericin B	0.25-0.5

**Table 7:** Quality control ranges for Candida albicans ATCC 90028, a reference strain used in antifungal susceptibility testing according to CLSI guidelines MIC, minimum inhibitory concentration; CLSI, Clinical and Laboratory Standards Institute

Antifungal Agent	MIC Range (µg/mL)	
	CLSI guideline	Findings in our study
Fluconazole	0.125-0.5	0.125-0.25
Voriconazole	0.004-0.016	0.006-0.008
Amphotericin B	0.125-0.5	0.135-0.195

## Discussion

*Candida* species is the most common cause of vulvovaginal candidiasis (VVC) in women, with about 75% of women experiencing at least one episode during their lifetime [30,31]. VVC is linked to considerable morbidity, as it represents the second most common cause of vaginitis globally, impacting millions of women annually across all social classes. The timely and accurate diagnosis of various *Candida* species is critical for the successful treatment of infections caused by this organism [32]. As the infectious mechanisms, as well as sensitivity to antifungal agents, vary from one *Candida* species to another, the proper identification of *Candida* species is essential for the better management of patients [33]. Research conducted across various regions shows that *Candida albicans* is the leading cause of VVC [34,35]. The high prevalence of *Candida albicans* can also be explained by its strong affinity for vaginal mucosal cells, which helps it to colonize and cause infection. In addition, *Candida albicans* is recognized among the *Candida* species for its ability to produce hyphae, which aids in tissue invasion [36,37]. While *C. albicans* is still the primary etiological agent of VVC, there has been a growing prevalence of non-albicans *Candida* species.

In the present study, the mean age is 29.6 years, which is similar to the findings reported in other studies in South Asia and worldwide. In a large prospective study of 500 women with culture-confirmed VVC in South India (Hyderabad), the mean age was 30.54 years (range: 18-64 years) [38]. In another Indian cohort of 211 clinically suspected VVC cases reported from Jawaharlal Institute of Postgraduate Medical Education and Research (JIPMER), Puducherry, culture-positive women ranged from 21 to 50 years with a mean age of ~33.1 ± 7.9 years [39]. A study in Nigeria reported the mean age for VVC to be 27.7 ± 7.8 years (patients’ age ranged from 16 to 45 years) [40]. Another study from Germany reported the overall mean age for women diagnosed with VVC to be 37.8 ± 15.5 years (based on 954,186 women with VVC diagnoses in gynecological records) [41]. A retrospective survey of VVC from Serbia reported the mean age of the participants with VVC to be 36.28 years [42]. A study from Turkey reported that the average age for all patients with VVC in an epidemiological sample was 38.4 years [43]. A study from Iran reported a cohort of patients with VVC with a mean age of 31.82 ± 10 years [44]. One feature that has been observed is that the mean age of VVC is lower in patients from developing countries and on the higher side in developed countries. VVC depends upon the patient’s hormonal condition and predominantly occurs in women of reproductive age. It is uncommon before menarche and after menopause, except in women receiving hormone replacement therapy [30].

Regarding marital status, our study revealed that the occurrence of VVC is more common in married women (85.42%) as compared to unmarried women (13.54%). This observation is also seen in a study from Ghana, which found a higher rate of VVC among married women compared to their unmarried counterparts [45]. An online survey involving 4,548 women from the USA also identified married women as being at greater risk of VVC [46]. In a report from Somalia, it was observed that 69.1% of women with VVC were married, while 30.8% were unmarried [47]. Many research studies include marital status because it often correlates with other behavioral or sociodemographic factors that can influence VVC risk, such as increased sexual activity and pregnancy.

In the present study, *Candida albicans* predominates with an occurrence in 75% of the cases, while the non-albicans *Candida* species accounted for the rest (25%). *Candida tropicalis* was the second most common pathogen, accounting for 15.63% of the patients, followed by *Candida glabrata* at 4.17%, which correlates with several other studies. *Pichia kudriavzevii* (*Candida krusei*) accounted for 3.13%, while *Candida parapsilosis* was reported at 2.08%. In a cross-sectional Indian study, *Candida albicans* was the predominant isolate (43.66%) in VVC cases. Among non-albicans *Candida* isolates, *Candida tropicalis* was the second most common isolate (38.03%), followed by *C. glabrata* (18.31%) [48]. This pattern was also similar to another study from India, which reported *C. albicans* as the predominant species (51.67%), and the next most common species was *C. tropicalis *(28.33%) [49]. Another study from Jaipur, India, reported *Candida albicans* prevalence at 54.54%, followed by *C. tropicalis* at 31.81% and *P. kudriavzevii* and *C. glabrata* at 9.09% and 4.54%, respectively [50]. In a study reported from Saudi Arabia on VVC, 68% were *Candida albicans*, followed by *C. tropicalis* (27%) and *C. glabrata *(2.7%), which is similar to the findings in the present study [51]. However, in a study from Uttar Pradesh, India, though *C. albicans* remained the leading species (59.8%) in VVC, *C. glabrata* was the next most common (15.3%), followed by *C. tropicalis* (9.8%) [52]. These findings were also similar to a study from Ethiopia, where *Candida glabrata* (15.3%) was the second most common cause of VVC, followed by *Pichia kudriavzevii* (14.1%), though *Candida albicans* was the predominant pathogen at 62.30% [53]. A study from Iran reported that *Candida albicans* was the most frequently isolated species (68%), followed by *Candida glabrata* (19.2%) and *Candida tropicalis* [20]. Other studies on VVC from Greece, Brazil, and Africa have reported that *Candida albicans* is predominantly involved in VVC, followed by *Candida glabrata, Candida tropicalis, Candida parapsilosis*,* *and *Pichia kudriavzevii (Candida krusei)* [11,14,15]. The same distribution can be seen in our study, though *C. tropicalis *is the most predominant non-albicans *Candida *species isolated in our study. The identification of *Candida *species is necessary for detecting strains such as *Pichia kudriavzevii*, which is intrinsically resistant to azoles, particularly fluconazole.

The phylogenetic analysis revealed species-level heterogeneity among the isolates examined in this study, with a predominance of *Candida tropicalis,* along with the detection of other clinically relevant species such as *C. albicans*, *C.* *glabrata, Candida parapsilosis*, and *Pichia kudriavzevii*. The distribution of isolates across multiple clades indicates considerable species diversity among the recovered yeast population.

Genetic diversity analysis plays an important role in understanding fungal epidemiology and guiding effective therapeutic strategies [54]. Fan et al. also emphasized that the knowledge of genetic diversity and resistance profiles among *Candida* isolates is essential for the effective management of vulvovaginal candidiasis [55].

The clustering of isolates with reference sequences confirms the presence of these species in the sampled population. The grouping of five isolates within the *C. albicans* clade suggests the presence of multiple lineages of this species, reflecting a heterogeneous population structure. Similarly, the occurrence of *C. glabrata*, *P. kudriavzevii*, and members of the *C. parapsilosis* complex further highlights the diversity of yeast species associated with the studied environment.

The relatively short terminal branches observed for most isolates indicate limited nucleotide divergence within species, whereas comparatively longer branches in a few isolates may reflect underlying genetic variability, possibly associated with microevolutionary processes or distinct haplotypic variants. Such diversity may contribute to the ecological complexity of yeast populations in the region and could have potential epidemiological and clinical implications.

Antifungal susceptibility testing was done for all 96 isolates against the commonly used antifungals in vulvovaginal candidiasis. VVC is usually treated with azoles such as fluconazole, which is given orally, often as a single dose, though longer courses may be required for persistent or recurrent VVC. VVC can also be treated with topical agents such as clotrimazole, miconazole, ketoconazole, and butoconazole. Polyenes such as amphotericin B, which is a powerful antifungal, are generally reserved for severe infections due to its toxicity [7]. The in vitro susceptibility testing of antifungal agents has become more critical as new antifungal drugs are introduced and resistant clinical isolates are increasingly identified. In the present study, *Candida albicans* showed higher susceptibility toward different antifungals commonly used in vulvovaginal candidiasis compared to the non-albicans *Candida* isolates, especially toward the azole antibiotics.

High fluconazole resistance was seen among all the different species of *Candida* isolated. In the present study, the sensitivity of *Candida albicans* toward fluconazole is 52.78%, while that of the non-albicans *Candida* species is 32.08%; this trend is also seen in several other studies from different parts of the world. In a clinical study of pregnant women with vulvovaginal candidiasis reported from Ghana, *C. albicans* isolates exhibited higher susceptibility to agents such as clotrimazole and nystatin compared with NAC species; specifically, fluconazole susceptibility was higher in *C. albicans* (44.7%) than in non-albicans *Candida *(21.1%) [56]. Mishra et al. from India reported that all *C. glabrata* isolates, 50% *C. tropicalis *isolates, and 12% *C. albicans* isolates were found to be resistant to fluconazole, thus showing the greater resistance of NAC toward fluconazole [57]. Bitew and Abebaw from Ethiopia also reported that *C. albicans* was the predominant species isolated and showed full susceptibility to several antifungals, whereas NAC species demonstrated higher resistance rates, particularly to fluconazole [14]. Similar observations were also reported by studies from Bulgaria, Greece, etc. [11,58].

Many of the non-albicans *Candida* species (such as *Candida glabrata, Pichia kudriavzevii*,* *and *Candida tropicalis*) exhibit intrinsic or decreased susceptibility to widely used azole antifungal agents. *Candida krusei* is inherently resistant to fluconazole, while *C. glabrata* frequently demonstrates dose-dependent susceptibility or resistance as a result of limited drug uptake. In comparison, *C. albicans* does not possess these innate resistance traits and is therefore generally more responsive to conventional antifungal treatments. Moreover, *C. albicans* typically has higher ergosterol dependence, making azole action more effective, and also has lower baseline efflux activity, leading to higher intracellular drug concentrations. Non-albicans *Candida *species produce more resistant biofilms, particularly on mucosal surfaces, which limit drug penetration and enhance resistance [59,60]. Among the azoles, the isolates showed higher susceptibility rates toward voriconazole; it is 88.89% for *Candida albicans* and 65.83% for non-albicans *Candida *species. González et al. [61] and Zeba et al. [62] also show similar findings, highlighting the superior efficacy of voriconazole over fluconazole and other azoles in the treatment of VVC.

In the present study, susceptibility to nystatin was 86.11% for *C. albicans* and 70.84% for non-albicans *Candida *species, while all the isolated strains were found to be susceptible to amphotericin B, thus indicating that they are more susceptible to the polyene group of antifungals. Rati et al. from Jaipur, India, report high amphotericin B sensitivity across *C. albicans* (75%) and non-albicans *Candida* (100% for *C. tropicalis, Pichia kudriavzevii, *and* C. glabrata*), while nystatin sensitivity was also very high for *C. albicans* (91.6%) and 100% for non-albicans *Candida* in this cohort [50]. Mbatudde et al. [63] and Kan et al. [64] reported susceptibility of 96% and 98%, respectively, to amphotericin B by *Candida* species. Though amphotericin B is broad-spectrum and more potent, its use is limited to the more severe infections due to high toxicity, but nystatin is more preferred in VVC because though it is not absorbed orally, it is highly effective locally. Lipid-based amphotericin B gel can be used topically in VVC with good results and fewer side effects [65]. The limitations of the present study are that analyzing a larger number of specimens over an extended period, first, would yield more detailed information on the distribution of different *Candida *species in the area and, second, would help us to give more conclusive evidence regarding the antifungal resistance pattern of the isolated *Candida *species; thus, further study with more clinical isolates needs to be done. Though the number of *Candida *species isolated in this study is limited, it is hoped that it will provide an idea of the trend of the occurrence of *Candida *species involved with VVC in this region and their antifungal susceptibility pattern.

## Conclusions

Clinical isolates of *Candida* species causing vulvovaginal candidiasis (VVC) should be accurately identified and their antifungal susceptibility patterns assessed to guide appropriate therapy and improve clinical outcomes. This study highlights the diversity of yeast species responsible for VVC in a tertiary care hospital in North-East India, with *Candida albicans* remaining the predominant pathogen, while an emerging presence of non-albicans *Candida* species was also observed. Some isolates demonstrated reduced susceptibility to commonly used antifungal agents, particularly azoles. Among the tested drugs, voriconazole showed comparatively lower resistance and may represent a potential therapeutic option alongside agents such as nystatin and amphotericin B. The continued surveillance of species distribution and antifungal susceptibility patterns, along with further regional studies, will be important to monitor evolving resistance trends and support the evidence-based management of VVC.
